# Supplementary material for: Are carbon emissions trading and green financial instruments synergistic? -Comprehensive quantitative research based on content analysis
Source: PLoS One. 2024 Mar 7;19(3):e0298601. doi: 10.1371/journal.pone.0298601 (PMC10919601; doi:10.1371/journal.pone.0298601)
Supplement: S1 Data — (ZIP) [file pone.0298601.s001.zip › Data/地级市Z_F工作报告.zip!/─┌├╔╣┼/╬┌║ú╩╨/└┤╫╘╣1⁄2╓┌║┼í╛┬φ┐╦╩2╛▌═°í┐_2019.docx]

各位代表：

现在，我代表市人民政府向大会报告工作，请予审议，并请政协委员和其他列席人员提出意见。

一、2018年工作回顾

2018年是贯彻党的十九大精神的开局之年，是改革开放40周年。一年来，在习近平新时代中国特色社会主义思想指引下，在自治区党委、政府和市委的坚强领导下，在市人大、政协的监督支持下，我们深入贯彻落实党的十九大和十九届二中、三中全会精神，持续打好三大攻坚战，扎实推进“五区”建设，凝心聚力、开拓奋进，全市经济社会平稳健康发展。全年地区生产总值增长13%，居全区首位；固定资产投资同比下降13.3%，居全区第3位；一般公共预算收入增长7.9%，居全区第4位；社会消费品零售总额增长6.3%，居全区第7位；城镇和农区常住居民人均可支配收入分别增长7.4%、9.5%，分别居全区第4位、第5位。

（一）聚焦三大攻坚战，发展短板加快补齐

稳控重大风险。采取债券置换、审计核销、国有资产盘活等措施，化解政府债务57.4亿元，完成年度化债任务的146%。引导企业优化股权结构、债务结构，企业债务水平有效降低。扎实推进社会矛盾纠纷排查化解攻坚行动，一批信访案件得到化解。严格落实安全生产“三个责任”[1]，安全生产形势总体平稳。聚力精准脱贫。精准制定帮扶措施，全面完成建档立卡贫困户脱贫工作。依托葡萄种植、光伏农业等产业，带动建档立卡贫困户户均增收6357元。贫困群众医保实行全额补贴，大病集中救治、低保救助实现全覆盖。狠抓污染防治。推进中央环保督察“回头看”反馈意见整改落实，实施污染防治项目244个。强化区域大气污染联防联控联治，中心城区空气优良天数达标率76.3%，高于自治区下达目标任务2.1个百分点。加强矿区环境综合整治，完成矸石、煤层自燃火点治理328处、矿山地质环境治理项目44个。推进矿区排土场集中连片治理，完成4家企业6个排土场整治。制定自治区首个矿区环境治理地方标准，建成自治区级绿色矿山4个，隆昌工贸骆驼山煤矿成为全区首个绿色矿山并写入自治区政府工作报告，被列入联合国开发计划署环境治理和可持续发展项目。完成电力企业超低排放改造，20蒸吨及以下燃煤锅炉全部淘汰，建筑工地达到绿色施工标准。加大水污染防治力度，三区污水处理厂建成运行，黄河流域水质持续好转。

（二）聚焦项目投资，发展基础逐步夯实

项目建设稳步推进。出台一系列促投资稳增长举措，实行领导干部包联重点项目责任制，全市重点项目开复工152个，开复工率达86.9%。强化民营企业帮扶，减免税费13.9亿元，帮助企业转续贷128.6亿元，电力多边交易为企业节约成本5.2亿元。招商引资加速加力。积极承接发达地区产业转移，实施招商引资项目122个，引进到位资金106亿元，湖北兴发有机硅、宝武炭材料产业园、科硕农药中间体等一批项目签约落地。园区振兴成效初显。市区两级投入3.4亿元，收储工业用地3392亩。争取跨盟市水权转让指标744万立方米，解决多家企业用水难题。投入10.4亿元，实施道路、供水等一批基础设施项目，园区配套更加完善。

（三）聚焦产业转型，新旧动能加速转换

创新引领能级提升。深化产学研合作，建成清洁煤基乙炔技术重点实验室、氯碱化工技术研究中心，国家高新技术企业达23家、自治区企业研发中心19家。航天十二院氢等离子体煤制乙炔军民融合项目进展顺利，信息化和工业化融合发展指数连续两年居全区首位。工业经济质效双提。规模以上工业增加值增长32.2%。煤焦化、氯碱化工产业链条不断延伸，集群优势进一步显现。引入北京建龙集团，对包钢万腾200万吨钢铁项目成功实施重组。精细化工产值增长62%，占规上工业产值比重达11%，集聚效应显著增强。战略性新兴产业加速崛起，中广核等7个光伏项目并网发电，中钰镁合金轮毂及板材、京运通新材料产业园等项目建成试生产。我市成为国家资源枯竭型城市转型年度考核七个优秀城市之一。现代服务业打开新局。服务业增加值占GDP比重达37%。乌海北站货场基本建成，中储南京智慧物流项目成为全区首家无车承运人[2]试点，乌海化工产业服务平台入选国家互联网与制造业融合发展试点。旅游业加快发展，乌海湖获评自治区级旅游度假区，龙游湾成为国家湿地公园。开展“内蒙古花季•乌海葡萄旅游季”等系列活动，全年接待游客352万人次，实现旅游收入76亿元，分别增长20%、17.5%，“来沙漠看海”旅游品牌影响力持续提升。金融业对实体经济支撑作用更加凸显，不良贷款余额和贷款不良率实现“双降”。推进乌海银行增资扩股，设立自治区首家旗县级股权交易工作站，我市首家孵化板企业在内蒙古股权交易中心挂牌，多层次资本市场加速形成。

（四）聚焦城乡融合，人居环境明显改善

城市功能持续完善。启动“多规合一”试点，完成新一轮城市总体规划修改，加紧编制生态修复、城市修补等16个专项规划。省道217线绕城段引线工程、甘德尔公铁立交桥开工，国道110乌海黄河特大桥主体合龙，荣乌高速乌海段建成。乌海机场旅客吞吐量达48.1万人次，同比增长5.9%。黄河海勃湾水利枢纽工程通过竣工验收。新改建街路14条，打通市民中心南路等9条断头路，改造人行道4.5万平方米。启动公交体制改革，购置新能源电动公交车100辆。新建改造供热、供气等管网156公里，环卫机械化清扫率达85%以上。建成便民市场21个，新增停车位近2万个，新改建公共厕所24个。乌达和海南净水厂主体完工，海勃湾北部净水厂进展顺利。投入近3亿元，实施乌海湖两岸等10个重点绿化项目，完成造林绿化1.5万亩。加快推进甘德尔山等一批亮化工程，城区夜景亮化水平全面提升。以创建全国文明城市和国家卫生城市为抓手，环境卫生治理等五大专项行动取得明显成效。强化交通秩序管控，主要交通路口通行效率提升30%。完成城市管理执法改革，实现市容环卫、市政公用、园林绿化统一执法。乡村振兴全面开局。启动农区人居环境整治三年行动，深入开展庭院环境治理，农区环卫清扫实现全覆盖。建成农区养老服务中心10个，农区公交通车率达92%。发展观光采摘、种植认养等休闲农业，启动建设鲜食葡萄等3个千亩基地工程，自然能源智慧农业项目进展顺利。健全龙头企业与农户利益联结机制，带动农区居民户均增收1.5万元。加快农区综合改革，集体产权制度试点改革、土地承包经营权确权登记颁证工作基本完成，土地草牧场“三权”分置改革顺利推进。严密防范非洲猪瘟，实现疫情“零发生”。

（五）聚焦改革开放，发展动力持续增强

全面深化改革纵深推进。相对集中行政许可权改革[3]试点、盐业体制改革完成，低碳城市试点等7项国家、自治区改革试点任务进展顺利。加快投融资体制改革，成功实施黄河能源科技集团20亿元债权转让，成为全区首个非上市民营企业债转股项目。持续推进“放管服”改革，编制“马上办、网上办、就近办、一次办”审批服务事项目录，市民网上办事大厅、掌上办事大厅上线试运行，34%的事项实现“马上办”，75%的事项实现“一次办”。清理各类证明事项134项，实现“32证合一”[4]。企业开办时限压缩到5个工作日，投资项目审批全部实现网上并联办理，工业项目审批时限压缩52.7%。市场主体、注册资本同比增长7.7%、37.5%，平均每天新增10户市场主体。实施质量强市战略，注册商标数同比增长38%。对外开放合作水平不断提升。成功举办中蒙煤焦化产业合作洽谈会，“乌蒙欧”班列常态化运营，乌海及周边地区货物通关实现“一站式服务”，对外贸易总额达7.4亿元，同比增长79.5%。与蒙古国达尔汗乌拉省建立友好地区关系，与意大利圣特拉姆、法国利布尔纳等友好城市合作不断加深。积极推进乌海及周边地区转型发展，36个区域合作项目取得实质性进展。

（六）聚焦民生保障，人民福祉不断增进

民生基础进一步夯实。城镇新增就业7758人，城镇登记失业率3.4%，零就业家庭保持动态清零。城镇职工基本养老、失业保险等覆盖面进一步扩大，连续14年上调退休人员基本养老金，城乡居民基础养老金实现“五连涨”。低保标准由635元提高到680元，困难供养人员集中、分散生活补助标准每人每月1600元、1260元，均居自治区前列。开工建设棚改住房8899套，基本建成10497套，超额完成年度任务。实施问题楼盘治理“百日攻坚”专项行动，群众高度关注的入住难、回迁难问题逐步解决。有序解决房产证历史遗留问题，为群众办理不动产权证2.2万户。社会事业全面进步。加快教育事业发展，幼儿园建设力度持续加大，海勃湾区六幼等5所幼儿园建成使用，全市标准园入园率提高到93%。普通高中本科上线率达82.6%，同比提高4.4个百分点。与兰州大学开展战略合作，乌海职业技术学院成为第三批国家级现代学徒制试点院校。乌海城乡社区大学、乌海老年开放大学挂牌。不断提高医疗服务水平，市人民医院晋级为三甲医院。深化医保付费方式改革，跨省异地就医结算医院达1.6万家，付费病种范围扩大到205个，群众就医负担进一步减轻。蒙中医院二期主体完工，市人民医院门诊综合楼项目启动。完善公共文化服务体系，举办文化惠民活动427场次，涌现出《乌海人家》等一批优秀文艺作品。成功举办国际帆板大赛、全国公路自行车联赛总决赛等重大赛事，城市影响力进一步提升。

（七）聚焦自身建设，行政效能稳步提升

深入推进“两学一做”学习教育常态化制度化，集中整治形式主义、官僚主义十种表现，政府系统作风建设持续加强。强化民主法治建设，主动接受人大法律监督、工作监督和政协民主监督，办理人大建议、政协提案291件，满意率98.6%。“七五”普法通过自治区中期验收。我市首部地方政府规章《城市市容和环境卫生违法行为处罚规定》发布实行。全面启动新一轮机构改革。严格落实中央八项规定及实施细则、自治区配套实施办法，“三公”经费支出同比下降18.1%。

国防建设、人民防空和双拥工作成效明显，妇女、儿童、青少年、老年人和残疾人权益得到较好保障，民族宗教、档案史志、外事侨务、海关、气象、防震减灾、食品药品安全、新闻出版、广播影视、无线电管理和红十字会等工作取得新进展。

各位代表，过去一年，我们认真贯彻落实中央、自治区和市委决策部署，迎难而上、集中攻坚，超额完成年度化债任务，全面完成建档立卡贫困户脱贫工作，顺利通过中央环保督察“回头看”的“检验考试”，三大攻坚战初战告捷。我们众志成城、奋力拼搏，积极应对有气象记录以来最大洪水，最大限度降低人民群众生命财产损失。我们成功举办第十届黄河明珠•中国乌海书法艺术节、承接“长三角”地区产业转移暨招商引资推介会等重大活动，广泛宣传乌海、推介乌海，城市知名度、美誉度进一步提高。实践充分证明：只要始终按照中央、自治区和市委部署要求，主动作为、埋头苦干，不折不扣抓好贯彻落实，各项工作就能顺利推进、干出成效；只要始终坚持发展第一要务，全力以赴转方式、调结构、稳增长，经济转型和城市转型就能实现稳中有进、进中提质；只要始终坚持以人民为中心的发展思想，全心全意为群众办实事、解难题，就能获得人民群众的信任和支持。

各位代表，一年来政府所做的工作，承载了自治区党委、政府的亲切关怀，体现了市委的正确领导，饱含了广大干部群众的辛勤汗水，凝结了社会各界的智慧力量。在此，我代表市人民政府，向广大干部群众，向关心支持乌海建设发展的社会各界人士，表示崇高的敬意和衷心的感谢！

需要向各位代表说明的是，受多种因素影响，我市固定资产投资、社会消费品零售总额增速虽然位于自治区前列，但完成情况仍略低于预期目标。同时，经济社会发展中还存在一些困难和问题：一是产业结构不够合理，粗放型发展方式尚未得到根本转变，新动能培育还需加强，转型发展任重道远。二是重大项目储备不足，固定资产投资增长乏力，财政收入增长压力较大。三是城市基础设施欠账较多，配套功能不完善，精细化管理水平有待提升。四是环保突出问题尚未得到根本解决，矿区环境综合整治任务繁重，大气污染防治形势依然严峻。五是民生领域还存在不少短板，特别是教育、医疗等优质资源不足、配置不均衡，与群众对美好生活的需要还存在较大差距。六是政府职能转变还不到位，少数干部责任意识和执行力不强，不敢担当、不愿作为、不善作为等问题依然存在。对此，我们将采取有力措施，切实加以解决。

二、2019年工作安排

2019年是新中国成立70周年，也是决胜全面建成小康社会的关键之年。做好今年工作，意义重大。今年政府工作总体要求是：以习近平新时代中国特色社会主义思想为指导，全面贯彻党的十九大和十九届二中、三中全会精神，统筹推进“五位一体”总体布局，协调推进“四个全面”战略布局，坚持稳中求进工作总基调，坚持新发展理念，坚持推动高质量发展，坚持以供给侧结构性改革为主线，坚持深化市场化改革、扩大高水平开放，加快建设现代化经济体系，继续打好三大攻坚战，着力激发微观主体活力，创新和完善宏观调控，统筹推进稳增长、促改革、调结构、惠民生、防风险工作，保持经济运行在合理区间，进一步稳就业、稳金融、稳外贸、稳外资、稳投资、稳预期，提振市场信心，增强人民群众获得感、幸福感、安全感，保持经济持续健康发展和社会大局稳定，为全面建成小康社会收官打下决定性基础，以优异成绩庆祝中华人民共和国成立70周年。

按照上述总体要求，今年我市经济发展主要预期目标是：地区生产总值增长6.5%左右，规模以上工业增加值增长8%左右，固定资产投资增长10%左右，一般公共预算收入增长6%左右，社会消费品零售总额增长6.6%左右，城乡居民人均可支配收入分别增长6%和7%左右，城镇新增就业6500人以上，其他约束性指标控制在自治区下达目标以内。

上述预期目标，既考虑到当前宏观经济形势错综复杂，不确定性因素增多，又考虑到我市现有发展基础，充分调动全市上下工作积极性，是市委、政府经过反复权衡、认真研究提出的。

各位代表，按照市委七届七次全体会议暨全市经济工作会议要求，2019年“五区”建设要在新兴产业培育、城乡产业融合发展、空气质量改善、项目审批服务、优质教育医疗资源供给上实现“五个突破”。我们将进一步抓重点、补短板、强弱项，更加注重投资、创新、改革、生态“四轮驱动”，厚植发展新优势、增强发展新动能，为推动经济高质量发展提供有力支撑。

强化投资拉动。投资是拉动经济增长的重要动力。要进一步创新政策举措，激发社会投资活力，大力推动民营经济繁荣发展，充分发挥社会投资对稳增长的关键作用。今年是西部大开发20周年，要紧紧抓住中央加大西部地区基础设施投资的政策机遇，在合理控制债务率前提下，持续加强政府投资，聚焦基础设施、民生改善等关键领域和薄弱环节，切实补齐发展短板。

强化创新驱动。创新是引领发展的第一动力。要着眼打造区域创新型城市，加大全社会研发经费投入力度，完善科技创新政策体系，推动产学研深度融合，加快科技成果转化落地应用。深化与国内信息产业龙头企业合作，以大数据、云计算技术“赋能”传统产业，推动工业经济转型升级。

强化改革促动。改革开放是推动高质量发展的活力之源。要持续深化“放管服”改革，进一步简化办事流程、压缩审批时限，全力破解企业和群众办事的难点、痛点和堵点，最大限度激发市场活力。深度融入国家“一带一路”和中蒙俄经济走廊建设，鼓励企业通过产能合作开拓国际市场，积极发展外向型经济。

强化生态撬动。良好生态环境是实现可持续发展的内在要求。要以壮士断腕的决心，坚决打好蓝天、碧水、净土三大保卫战，推动生态环境实现明显好转。充分发挥环保倒逼机制作用，通过环境容量、能源消费总量和污染物总量控制，引导企业淘汰落后产能、实施整合重组，实现绿色发展、低碳发展、集约发展。

重点抓好以下七个方面工作：

（一）坚持三大攻坚战稳扎稳打，坚决守住发展底线

打好防范化解重大风险攻坚战。统筹做好债务化解工作调度和摆布，综合采取各类措施，确保全市综合债务率持续下降。新上政府投资项目严格进行债务风险评估，做到量力而行。密切关注企业债务风险，积极协调金融机构通过债转股等方式处置不良贷款，确保不发生系统性风险。牢固树立过紧日子思想，严控“三公”经费预算，市、区两级财政原则上按照不低于5%的比例压减一般性支出。学习运用新时代“枫桥经验”[5]，完善矛盾纠纷排查化解机制，全力维护社会稳定。严格落实安全生产责任制，健全应急管理和防灾减灾体制机制，坚决遏制重特大事故，推进城市安全发展。

打好精准脱贫攻坚战。强化农区贫困人口动态监测管理，建立健全稳定脱贫长效机制，进一步巩固提升脱贫攻坚成果。切实解决贫困“边缘户”缺乏政策支持等问题，防止返贫和产生新的贫困。大力推进产业扶贫，进一步把贫困户吸附在产业链上。落实好兜底保障、就业扶贫等各项政策，实施好安全饮水、危房改造等扶贫工程。切实加强健康扶贫，强化大病保险、医疗救助等“五重医疗保障”，避免因病致贫、因病返贫。注重扶贫和扶志、扶智相结合，激发贫困群众内生动力和自我发展能力。

打好污染防治攻坚战。坚决抓好中央环保督察及“回头看”反馈意见、自治区生态环保大检查反馈问题整改落实，切实提高生态环境质量，力争中心城区空气质量优良率达到77.6%以上。打好蓝天保卫战。制定《乌海市绿色矿山建设总体规划》，实施27个矿山地质环境综合整治项目，完成35个排土场、6个着火点治理，新增3个自治区级绿色矿山，促进矿区环境持续好转。加快西鄂尔多斯国家级自然保护区企业退出步伐，推动保护区生态环境修复。集中治理工业污染，重拳打击超标排放企业，完成焦化、水泥等重点行业特别排放限值提标改造，工业企业大气污染物年日均值浓度达标率力争达到95%以上。强化面源污染治理，开展烟煤散烧、道路扬尘等专项整治，抓好乌海湖清淤和库区扬尘治理。强化乌海及周边地区大气污染联防联控联治，定期开展联合执法，健全重污染天气联合预警机制。打好碧水保卫战。全面落实河长制，加强黄河乌海段水体监管。推动三区污水处理厂稳定运行，进一步提高中水回用率。推进违规取用地下水专项整治，积极争取跨盟市水权转让指标。打好净土保卫战。强化土壤污染防治，开展农用地、重点企业用地土壤污染状况调查，严格监管各类土壤污染源。实施“清废行动”，加大固废、危废等处置力度，确保土壤环境安全。

各位代表，打好三大攻坚战是全面建成小康社会必须跨越的关口。我们要按照中央和自治区统一部署，坚持底线思维，集中优势兵力，坚决打好这几场关键性的战役，不获全胜、决不收兵!

（二）坚持项目建设为重为要，夯实经济发展基础

狠抓项目建设。开展“优环境强服务促落实项目建设年”活动，建立健全项目引进—前期—落地—建设—投产“一条龙”协作服务机制，力促项目早开工、早达产、早见效。实行项目审批全程代办帮办、容缺受理等措施，为企业提供“保姆式”服务。创新重点项目包联推进机制，建立评价、考核制度，切实提高包联实效性。全年计划实施重点项目185个，完成投资180亿元。启动“十四五”规划编制前期工作，提前做好重点项目储备。狠抓招商引资。积极承接长三角、珠三角、京津冀产业转移，通过产业链招商、以商招商、委托招商等方式，引进一批发展前景好、带动能力强的大项目好项目，培育一批成长性高、竞争力强的隐形冠军、瞪羚企业。建立招商引资目标管理考核体系，切实提升招商引资实效。坚持择商选资，严把项目引进关口，提高准入门槛，不能“捡到篮子里都是菜”，坚决不要“带灰的GDP”。力争全年引进到位资金增长6%以上。企业新上项目或重大技术改造项目，适用招商引资政策。狠抓园区振兴。实施“筑巢引凤”行动，以财政资金撬动社会资本，加快推进低碳园区污水处理厂等园区基础设施项目。加大“腾笼换鸟”力度，坚决出清“僵尸企业”，为转型项目落地腾出空间。制定完善“飞地经济”[6]政策，实行项目异地落户利益共享机制。坚持“以亩产论英雄”，建立园区高质量发展考核指标体系，提高投资强度、单位能耗等考核权重。狠抓民营经济。健全领导干部联系重点民营企业机制，对民营企业发展重点难点问题，实行台账管理、精准帮扶、跟踪问效。加强政金企对接，充分发挥助保贷资金池、政策性担保机构作用，切实解决企业融资难、融资贵问题。制定出台促进民营经济发展政策措施，充分激发民营经济发展活力。认真梳理兑现招商引资优惠政策，专项治理机关事业单位和国有企业拖欠民营企业资金问题。严肃整治损害营商环境的慢作为、不作为、乱作为现象，不断提升民营企业满意度和获得感。

各位代表，抓项目就是抓投资，就是抓发展。我们要以项目落实为抓手，以项目落地看担当，在加强招商引资、完善基础设施、优化营商环境上狠下功夫，实施一批打基础、利长远、强支撑的重点项目，为全市经济社会健康发展增添不竭动力！

（三）坚持产业发展提质提效，全力构建现代经济体系

提升科技创新能力。以科技创新驱动产业转型，举办首届乌海化工产业科技论坛，加强与兰州大学、天津大学等高等院校产学研用合作。探索设立科技创新引导基金，鼓励企业加大工艺技改、节能环保等方面投入，推动工业经济提档升级。在北京、上海、兰州等地建立联合实验室和科研成果转化中心，加快院士专家工作站建设，推动一批先进适用技术实现异地孵化、乌海转化。推进航天十二院氢等离子体煤制乙炔、天宇军用领域新材料等项目，促进军民融合不断深化。依托驻外人才工作站，大力招才引智，带动高端人才加速集聚。围绕产业转型发展需求，大力引进和培养一批专业技术人才。优化人才发展环境，认真落实人才引进政策，确保引得进、留得住、干得好。

优化升级传统产业。深挖现有项目潜力，加快盘活存量，力促华油天然气LNG等重点项目尽快复产，确保包钢万腾200万吨钢铁顺利达产。围绕“两大基地”建设，推动焦炉煤气综合利用、煤焦油深加工、粗苯精制等产业链不断延伸，力争宝武集团炭材料产业园开工建设，着力打造煤焦化产业集群。推动洪远聚乙烯复工，确保中联化工一期50万吨PVC、君正化工PVC扩建等项目落地开工，着力打造氯碱化工产业集群。抓好亚东、佳瑞米等一批医药农药中间体项目，推动工业向精细化、终端化方向发展，着力打造精细化工产业集群。

培育壮大新兴产业。完善项目手续办理、金融支撑、土地使用等扶持政策，推动新能源、新材料、装备制造、信息技术等战略性新兴产业发展提速、比重提升。加快恒健三元正极材料、懿福新能源汽车、奥立再生玻璃纤维二期、亿邦大数据产业园等项目建设，力促协鑫等3个光伏项目并网发电，着力打造新能源、新材料产业集群。编制现代能源经济发展规划，开展氢经济示范城市[7]前期工作。

繁荣发展现代服务业。研究出台推动物流业发展等相关政策，围绕主导产业做好配套服务。大力发展平台经济，充分发挥西北化学网、鸿达大宗物资交易中心等平台作用，进一步提升对周边地区乃至整个西部的辐射力、带动力。依托“山、水、沙、城”特色景观优势，支持兴泰集团开发建设环乌海湖旅游项目，积极创建国家级旅游度假区。推进甘德尔山生态文明景区等重点景区建设。完善沿黄旅游道路、休闲驿站、停车场等基础设施，优化旅游线路，实现旅游收入80亿元以上。继续推动乌海银行增资扩股，鼓励支持企业上市，大力发展保理、融资租赁、融资担保等金融新业态，着力打造区域金融服务中心。

各位代表，加快新旧动能转换是当前我市经济发展的首要任务。我们要下定决心、保持定力，在改造提升传统动能、培育壮大新动能上聚焦发力，打造一批规模优势明显、带动性强的产业集群，迈出乌海高质量发展新步伐！

（四）坚持城乡发展互融互促，打造生态宜居城市

完善城市功能。实施规划提质工程，着眼生态修复、城市修补，统筹经济社会发展总体规划和国土、城乡建设等专项规划，扎实推进“多规合一”[8]。实施道路畅通工程，加快包银高铁乌海段、省道318线乌海湖东段、省道37线前期工作，抓好省道217线绕城段、甘德尔公铁立交桥等项目建设，确保国道110乌海黄河特大桥顺利通车、乌海客运枢纽站建成运营。新改建滨河大道等城区道路17条，打通团结路等一批“断头路”。新购置一批新能源电动公交车，完善公交运输网络，积极创建国家公交都市建设示范城市。实施市政提升工程，完善供水、供热管网等基础设施，新建绿色公厕120座。加快海勃湾北部净水厂建设，乌达、海南净水厂建成使用，切实保障群众饮水安全。开展泄洪道清理整治专项行动，完善市政排水系统，增强城市综合防汛能力。实施百姓安居工程，加大棚户区改造力度，开工建设保障性住房8042套，基本建成6587套。聚焦问题楼盘治理、不动产权证办理、物业服务管理，全力保障群众“住得上、住得好”。实施管理优化工程，精准治理交通组织、街巷卫生等方面存在的问题，不断提升市民文明素质，积极创建全国文明城市。完善城市管理综合执法机制，推动城管执法规范化、程序化、法制化。实施环境美化工程，大力推进生态修复和国土绿化，抓好乌海湖东岸绿化、植物园改造提升等重点项目建设，完成造林1万亩以上。通过拆迁建绿、择空补绿等方式，增强城市“绿肺”功能。完成乌海湖大桥等一批亮化工程，优化小街小巷小区照明系统，进一步提升城市亮化品质。

推进乡村振兴。让特色农业强起来。编制乡村产业振兴规划，健全完善政策扶持体系。坚持品牌兴农、绿色兴农、科技兴农，加强高标准农田建设，推广葡萄标准化种植，新增葡萄种植面积2000亩。充分发挥龙头企业带动作用，打造精品葡萄酒庄，提升世界沙漠葡萄酒品牌知名度和影响力。依托农业产业园等重点项目，建设集观光采摘、特色养殖、度假体验等于一体的田园综合体，促进三次产业融合发展。鼓励设施农业发展，进一步丰富市民“菜篮子”。让农区环境美起来。加强农区人居环境综合整治，优化街巷、庭院环境，抓好畜禽粪污资源化利用，力争生活垃圾处理率达到100%、卫生厕所普及率达到80%以上。加快“四好农村路”[9]建设，新改建农区公路60公里以上。推动广电网络基本公共服务入户，农区光纤网络覆盖率达到90%以上。充分发挥掌上办事大厅等服务平台作用，构建城乡政务服务一体化体系，真正实现信息多跑路、农区居民少跑腿。按照中央、自治区统一部署，深入推进“大棚房”[10]问题清理整治。让农区居民富起来。完善农企利益联结机制，培育葡萄、蔬菜等产业化联合体，带动村集体经济发展和农区居民增收致富。深化土地草牧场“三权”分置改革，盘活土地资源，增加农区居民财产性收入。推进农区集体产权制度改革，激发乡村振兴内生动力。

各位代表，城乡融合、互动发展是全面建成小康社会的必然要求。我们要按照“以城带乡、以乡促城、融合发展”的工作思路，统筹推进城区和农区规划建设管理，推动基础设施同步提质、面貌同步改善、居民生活同步提升，着力打造城乡融合先行区！

（五）坚持改革开放落地落细，激发市场主体活力

推进改革攻坚。深化供给侧结构性改革，着力在“巩固、增强、提升、畅通”上下功夫，促进实体经济蓬勃发展。全力抓好低碳城市等改革试点任务，以点带面推动改革走向纵深。加大“放管服”改革力度，投资项目审批办理时限压缩到100个工作日内，减少审批材料60%以上，100个高频事项实现“最多跑一次”。完善“互联网+政务服务”，力争“网上办”事项达到70%以上。强化事中事后监管，加大“双随机”检查力度。深化国有企业改革，确保国有资产保值增值，实现从管企业向管资本转变。推进财税体制改革，加快市区财政事权与支出责任划分改革。抓好投融资体制改革，强化融资平台和各类基金管理。完成市、区两级政府机构改革，加快转变政府职能。大力推进事业单位改革。完善信用联合奖惩机制，积极创建全国社会信用体系示范城市。扩大开放合作。主动融入自治区向北开放战略，依托策克、甘其毛都和乌力吉口岸，着力打造蒙古煤大宗产品交易平台，把通道经济变为落地经济。依托乌海海关、国际陆港，提升“乌蒙欧”班列开行频次，建设保税仓，进一步扩大对外贸易。抓住自治区打造沿黄生态经济带和推进乌海及周边地区转型发展的重大机遇，积极推动与周边园区基础设施互联互通、产业上下游协作，形成差异化、协同化发展格局。

各位代表，改革开放是破解转型发展难题的关键一招。我们要紧紧围绕建设投资贸易首选区，以改革增活力、以开放添动力，再造发展新优势、拓展发展新空间，着力打造自治区西部新的经济增长极！

（六）坚持发展成果共建共享，满足群众美好生活需要

推进教育提质发展。深入实施第三期学前教育行动计划，新改建5所幼儿园，进一步提高普惠性幼儿园比例。加强与高等院校教研合作，深化教育信息化技术应用，不断提升教育教学水平。实施教师队伍素质提升工程，大力培养一批青年骨干教师，引进一批名优特教师。引进衡水中学优质教育资源，成立乌海市衡水实验中学。完善教育基础设施，抓好市一中新校区、市十中教学楼建设。加快发展职业教育，筹建内蒙古航空旅游职业学院，促进产教融合、校企合作。统筹推动民族教育、特殊教育有序发展。

加快建设“健康乌海”。深化“三医联动”，开展现代医院管理试点，稳步推进医保支付方式改革，进一步降低群众就医负担。完善基层医疗卫生服务网络，打造“一刻钟”卫生普惠服务圈。实施新一轮改善医疗服务三年行动计划，加强与国内知名医学院校和医疗机构合作，不断提升医疗技术水平。开工建设市人民医院门诊综合楼，建成蒙中医院二期。振兴发展蒙医中医药事业。深入开展爱国卫生运动，积极创建国家卫生城市。

提高社会保障水平。把稳就业放在突出位置，促进高校毕业生、退役军人等群体多渠道就业创业，城镇登记失业率控制在自治区下达目标以内。采取担保贷款、创业辅导等措施，鼓励引导大学生自主创业。推进全民参保登记计划，扩大跨省异地就医直接结算范围，满足群众多层次就医需求。加强欠薪源头治理，确保农民工工资如期足额支付。加大社会救助力度，切实保障低保、特困供养人员等困难群众基本生活。鼓励社会资本参与养老机构建设运营，加快养老服务产业发展。

繁荣发展文体事业。围绕新中国成立70周年等主题，组织开展各类庆祝活动和文化惠民展演。推进乌兰牧骑事业发展，为群众提供面对面、接地气的公共文化服务。办好世界沙漠葡萄酒文化节等重大活动，大力发展书法等文化产业。广泛开展全民健身活动，推动公共体育场馆免费向社会开放。举办乌海国际马拉松等一系列体育赛事，带动体育产业发展。

完善社会治理体系。健全社区服务功能，实行网格化管理、社会化服务，推动社会治理重心向基层下移。扎实开展“七五”普法宣传，切实提高全社会法治意识。强化社会治安防控，深入推进扫黑除恶专项斗争，加快平安乌海建设。健全食品药品监管机制，积极创建国家食品安全示范城市。

各位代表，人民对美好生活的向往就是我们的奋斗目标。我们要不断加大民生投入，解决好老百姓最关心、最直接、最现实的利益问题，全力以赴补齐民生短板，让广大群众获得感更充实、幸福感更真实、安全感更踏实!

（七）坚持自身建设从严从实，打造为民务实清廉干部队伍

新的一年，我们承载着更大期盼，肩负着更大责任。我们将不断加强自身建设，做到政治过硬、责任过硬、能力过硬、作风过硬，奋力开创各项工作新局面。依法行政明权责。依法接受人大法律监督、工作监督，自觉接受政协民主监督，重大事项主动向人大报告，重点工作主动与政协协商，高质量办理人大建议和政协提案。支持监察委、法院和检察院工作，强化审计监督。严格按照法定权限履行职责、行使权力，运用法治思维解决问题、化解矛盾，依据法定权责科学决策、推动发展。修订完善市政府工作规则，提升政府工作规范化制度化水平。清正廉洁树形象。切实履行党风廉政建设主体责任和“一岗双责”，把廉洁从政要求贯穿于政府工作全过程、各环节，严厉查处各类不正之风和腐败问题。强化权力运行制约监督，健全工程建设、土地出让、政府采购等重点领域廉政风险防控机制，从源头上预防和遏制腐败行为，保持政府廉洁本色和良好形象。勤政为民办实事。着力解决好群众高度关注的社会民生问题，以实际行动积极回应群众期待，让群众得到更多实惠。充分发挥“12345”市民服务热线作用，打造一座连接政府和群众的“连心桥”。扩大政务公开范围，充分保障群众的知情权、参与权和监督权。改进作风抓落实。严格执行中央八项规定及实施细则、自治区配套办法，坚决破除形式主义、官僚主义。深入开展“不忘初心，牢记使命”主题教育，推进“两学一做”学习教育常态化制度化。大力倡导“说了就办、定了就干、干就干好”的实干精神，善打攻坚战、敢啃“硬骨头”，不断提升政府执行力和战斗力。集中整治“懒官、庸官、乖官、巧官”四官问题，严肃查处不作为、慢作为、假作为、乱作为。下大力气精文减会，把会场变现场，纠正凡事留痕的做法，把更多时间和精力用在抓落实上。发扬“钉钉子”精神，一锤接着一锤敲，一件接着一件干，确保各项工作落到实处、见到实效。

各位代表，当前，乌海正处于创新发展、转型发展的关键期。做好2019年政府工作，任务艰巨，责任重大。让我们更加紧密地团结在以习近平同志为核心的党中央周围，高举习近平新时代中国特色社会主义思想伟大旗帜，在自治区党委、政府和市委的坚强领导下，拼搏实干，锐意进取，为决胜全面建成小康社会，谱写新时代乌海高质量发展新篇章而努力奋斗，以优异成绩庆祝新中国成立70周年!
